# ‘Managing pieces of a personal puzzle’ — Older people’s experiences of self-management falls prevention exercise guided by a digital program or a booklet

**DOI:** 10.1186/s12877-019-1063-9

**Published:** 2019-02-18

**Authors:** Beatrice Pettersson, Maria Wiklund, Rebecka Janols, Helena Lindgren, Lillemor Lundin-Olsson, Dawn A. Skelton, Marlene Sandlund

**Affiliations:** 1 Department of Community Medicine and Rehabilitation, Physiotherapy, Umeå University, Umeå, Sweden; 2 Department of Community Medicine and Rehabilitation, Occupational Therapy and Department of Computing Science, Umeå University, Umeå, Sweden; 3 Department of Computing Science, Umeå University, Umeå, Sweden; 4 School of Health and Life Sciences, Glasgow Caledonian University, Glasgow, UK

**Keywords:** Accidental falls, Aged, Exercise, Qualitative research, eHealth, Digital health, Self-management, Falls prevention, Intervention, Behaviour change

## Abstract

**Background:**

Exercise is effective in order to prevent falls in community-dwelling older people. Self-management programs have the potential to increase access and reduce costs related to exercise-based fall prevention. However, information regarding older people’s views of participating in such programs is needed to support implementation. The aim of this study was to explore older people’s experiences of a self-management fall prevention exercise routine guided either by a digital program (web-based or mobile) or a paper booklet.

**Methods:**

This qualitative study was part of a feasibility study exploring two completely self-managed exercise interventions in which the participants tailored their own program, guided either by a digital program or a paper booklet. Individual face-to-face semi-structured interviews were conducted with a purposeful sample of 28 participants (18 women), mean age 76 yrs. Qualitative content analysis was used to analyse the data.

**Results:**

Self-managing and self-tailoring these exercise programs was experienced as ‘Managing pieces of a personal puzzle’. To independently being able to create a program and manage exercise was described in the categories ‘Finding my own level’ and ‘Programming it into my life’. The participants experienced the flexibility and independence provided by completely self-managed exercise as positive and constructive although it required discipline. Furthermore, different needs and preferences when managing their exercise were described, as well as varying sources of motivation for doing the exercise, as highlighted in the category ‘Defining my source of motivation’. The category ‘Evolving my acquired knowledge’ captures the participants’ views of building their competence and strategies for maintenance of the exercise. It describes a combined process of learning the program and developing reflection, which was more clearly articulated by participants using the digital program.

**Conclusions:**

This study provides new knowledge regarding experiences, preferences and motivations of older people to engage in home-based self-managed fall prevention exercise. They expressed both a capability and willingness to independently manage their exercise. A digital program seems to have strengthened the feeling of support while creating their own exercise program and tailoring it to their preferences and circumstances, which might therefore create better opportunities for adoption and adherence in the long term.

## Background

Falls present the most common cause of unintentional injuries among older people [1, 2] and often lead to psychological consequences such as fear of falling and avoidance of activity [3, 4]. Exercise, as a standalone intervention, has been shown to reduce falls and fear of falling among community-living older people [5–7]. To improve effectiveness, components of balance and strength training as well as a higher dose of exercise have been identified as important [8]. The increasing population of older people present an ever-growing challenge of increased falls, and so it is imperative that research identifies ways to engage them in evidence-based, accessible and cost-effective falls and injury prevention programs.

Unfortunately, adherence to falls prevention programs is often poor over time, with only approximately 52% of community-dwelling older people still active after one year [9]. Older people have different needs and desires in relation to their motivation and intention to undertake fall prevention programs [10]. Due to its social component and peer support, group exercise is often emphasized as a way to increase exercise motivation [11]. Although social interaction is an important facilitator for many, group exercise can also pose many barriers, such as lack of transportation, time, costs or concerns about ability level compared to others [12–14]. Indeed, it has been reported that many older people prefer exercising at home, where they also have the opportunity to integrate exercise into their everyday activities [15], a strategy which have been shown to reduce falls [16].

Through self-management, the older person takes an active role in the development, tailoring of and engagement in their exercise routines [17]. It has, therefore, been suggested that adherence would be improved by increasing their exercise self-efficacy [10]. Self-management approaches are most commonly utilized in management of chronic conditions [17] and have been associated with improved outcomes when combined with behavioural change strategies [18, 19]. Home exercise programs for older people are often prescribed by an expert and the active role of the older person to select and progress their training is often limited.

By the use of digital programs it is possible to support the older person in an active role of self-management. It is also possible to include a variety of behavioural change strategies to facilitate maintenance of the exercise such as action planning or self-monitoring [19, 20]. It has previously been shown that both adherence and physical functioning improved when older adults were given a digital exercise program with individual and social motivational components, compared to a home exercise booklet [21]. Digital health is emerging as an effective way to distribute self-management exercise and can potentially increase access and reduce costs, while promoting an active lifestyle for older people with different diagnoses [22, 23]. However, there is a lack of studies investigating fall prevention exercise programs that the older person can use and progress entirely on their own with only a short introduction.

To best support implementation of completely self-managed fall prevention exercise it is important to investigate the older participants’ experiences of using such programs in different formats. The aim of the present study was to explore older people’s experiences of a self-management fall prevention exercise routine guided either by a digital program or a booklet.

## Method

### Study design

This qualitative study, where face-to-face individual interviews were conducted, is part of a feasibility study (ClinicalTrials.gov ID: NCT02916849) comparing two home based self-management exercise programs: a digital program and a paper booklet (as described below). Participants’ experiences of self-management exercise and experiences related to the different programs were analysed using qualitative content analysis [24].The study was approved by The Regional Ethical Review Board in Umeå (Dnr 2016/106–31). This is the first study presented from the feasibility study and further results will be presented elsewhere.

### Study context and background

First, a self-management digital exercise program (Safe Step v1, web-based or mobile) with integrated strategies for behavior change support was developed by a multi-disciplinary team of researchers in collaboration with older people, guided by a participatory research approach [25]. The digital program was influenced by the Otago home exercise program [26] but was further expanded with both easier and more challenging exercises. Second, a modified version of the Otago Home Exercise Booklet [27] was developed by researchers in our research team. Finally, the two programs were compared in a feasibility study.

The feasibility study was conducted in Umeå, northern Sweden (latitude 63.8° north) where snow or ice lays on the ground during the main part of winter (Dec-Mar). Recruitment took place either at a health care centre, by health care staff, or in senior citizens organisations by members of the research team. The participants were presented with both the digital program and the paper booklet and were free to select their preferred program. Participants from the Senior citizens organisations who chose the digital program were, after recruitment, presented with an additional choice of attending a group peer-mentor meeting once a month. In total, 67 participants were included, 29 chose the digital program (of which 12 chose the mentor group) and 38 chose the paper booklet. Inclusion criteria for the feasibility study were 70 years or older with self-reported impaired balance, to be able to rise from a high chair, and to be able to stand without support. A previous fall was not necessarily required for inclusion. Exclusion criteria were a progressive disease that impaired mobility, doing intense exercising more than 3 h/week or showing signs of severe memory problems. Any memory problems were determined not to be severe if the participants were able to effortlessly answer questions and discuss the details of the study during the inclusion procedures.

### Exercise programs

The two exercise programs, the digital and the paper booklet, were both completely self-managed. This meant that the older person could tailor how, when and where to use the program and progress the training entirely on their own. Both programs consisted of repositories of evidence-based strength and balance exercises and were free of charge. The digital program was delivered through a computer, smartphone or tablet and the other program by a paper booklet (Table 1). In the digital program the exercises, presented in video format, were organized into 10 predetermined groups consisting of exercises with a main focus to increase strength for different lower-limb muscle groups (5 groups), improve balance (2 groups) and gait/step (3 groups). For each group the participant was able to choose between various exercises to find an alternative suitable for their ability. In the booklet the exercises were organized into strength or balance exercises and also in three different levels of difficulty where the participant was asked to choose 10 suitable exercises, five balance and, five strength exercises. All participants were asked to choose exercises that they felt would be challenging but not too hard. Meaning for balance exercises that they should feel unstable but not losing their balance and falling, and for strength exercises that they should feel strain in the muscles and able to follow the recommended repetitions. They were also recommended to select a new exercise whenever they felt the current exercise became too easy or too difficult.

**Table 1:** Overview of the exercise programs

Characteristics	Digital program	Booklet
Exercise presentation	Video clips with oral instructions. A repository of exercises were organized into 10 groups: 5 groups of exercises to increase lower-limb muscle strength, 2 groups to improve balance and 3 groups combining strength and balance in gait and step exercises. Each group contained exercises on different levels of difficulty.	Drawings with written instructions. A repository of exercises were organized into strength and balance exercises, each part divided in 3 different levels of difficulty.
Behaviour change support	Written feedback from virtual physiotherapist, activity planning, monitoring progress (i.e. charting activities and frequency of exercise).	None
Exercise diary	Integrated in digital program	Paper based returned monthly
Safety instructions	Adapted advice for every exercise in the videos and optional advice for every exercise group. General safety information for everyday life.	A page in the booklet presented general safety instructions while doing the exercises.
Other	Examples of exercises integrated into everyday activities and tips on how to do exercises outdoors.	None

Before starting their self-management program, the study participants attended a group meeting for a short introduction to falls and falls prevention and an introduction to the exercise program of the participants’ choice. The introduction included an explanation of the main structure of the program, instructions on how to select exercises, how to fill out the exercise diary, safety aspects during the sessions at home and possibilities to alter exercises. Physical assessment and self-administered questionnaires were completed. All participants were requested to exercise for at least 30 min three days a week. After the introduction meeting the participants were asked to create their own program at home and continue to exercise for four months. Almost half of the digital group chose to attend the peer-mentor meetings once a month. All participants received a phone call from a researcher after 2–3 weeks and optional support was provided through a hotline telephone number throughout the study.

### Participants

Participants were recruited to this qualitative study at the post-assessment meeting of the feasibility study, by a member of the research group. A strategic selection of men and women was made to get a heterogeneous group and based on the place of recruitment, program used and how much they had exercised during the four month intervention. We asked more participants from the digital group to participate since we wanted to include both those who did and did not attend the peer-mentor meetings. Thirty participants from the feasibility study were approached and 28 accepted (Table 2).

**Table 2:** Descriptive characteristics of the two exercise groups in this qualitative study

Characteristics	Digital program *n* = 17	Booklet *n* = 11
Age yrs., mean (min-max)	76 (71–91)	77 (73–85)
Women, *N* (%)	10 (59)	9 (82)
Access to smart phone/tablet, *N* (%)	14 (82)	6 (55)
Access to computer, *N* (%)	14 (82)	7 (64)
Attended peer-mentor meetings, *N* (%)	6 (35)	NA
Activity level SGPALS (score 1–6), median (Q1-Q3) Summer Winter	4 (3–4) 3.5 (3–4)	3 (3–3,5) 3 (3–4)
Household situation, *N* (%) Living together Living alone	12 (71) 5 (29)	8 (73) 3 (27)

*SGPALS* Saltin-Grimby Physical Activity Level Scale [40], *Q* quartile, higher = more active. Summer months defined as Jun-Aug, winter as Dec-Mar

### Data collection

Individual semi-structured interviews were conducted with 19 women and 9 men and lasted between 19 and 80 min, the average being around 45 min. Two of the interviews were conducted with spouses using the same program. Prior to the interviews, an interview guide was developed with open-ended questions and was structured around five themes identified as relevant to the aim: How the exercise programs were used (where, when and what they had done); perceived effects of the exercise in terms of safety, structure and content of the programs; personal motivators to do the exercise; and maintenance of the exercise. The interviews started with the interviewer asking the participant to describe how they usually performed their exercise. To facilitate a feeling of comfort the participants had the opportunity to choose if the interviews would be held at their home, at the university or at a library. The interviews were carried out by one of two physiotherapists with long experience of interviews or by one of four physiotherapist students, two of which were under supervision or two with previous experience of conducting interviews, all women. Each interview was audio-recorded, transcribed verbatim and included in the analysis.

### Data analysis

Data was analysed using inductive qualitative content analysis that focuses on similarities and differences within the material expressed through both manifest and latent levels of abstraction [28]. The first author had the main responsibility for the analysis. First a naïve reading of the material was done to get an initial understanding and thereafter each interview was read on a more profound level. Meaning units were identified based on their relevance for the aim of the study and were further condensed after which codes, representing the core of the condensed units, were created. Initially, a few of the interviews were coded and the meaning units and codes were deliberated and agreed upon by three of the authors (BP, MS and LLO). The codes were thereafter sorted into subcategories and categories based on an interpretation of the manifest content by the first author and were on several occasions discussed by all authors to ensure confirmability [24] (Table 3). Finally, a theme representing the common ground and latent content of the material was formulated. Throughout the whole process researchers with different competence and perspectives (physiotherapy, exercise physiology, computing science, human-computer interaction) were involved in the analysis to triangulate and thereby increase credibility and dependability of the results [24]. All researchers were PhDs and one a PhD-student (BP). To further strengthen the credibility, the results were also presented during a PhD-research seminar and an international research meeting, where feedback was received from those not involved in the study. The researchers and the participants were not known to each other before the start of the feasibility study. The qualitative data software Open Code 3.4 was used to facilitate the analyses [29]. To ensure transferability [24] the methods and results (including quotations) were described in detail - according to recommendations for qualitative interviews (COREQ) [30].

**Table 3:** Examples from the process of analysing the data using qualitative content analysis

Examples of meaning units	Examples of condensed meaning units	Examples of codes	Sub-categories	Category
I’ve done the instruction so many times that it’s in my head now so to speak.	It is in my head after doing it several times	It is in my head	Learning the program	Evolving my acquired knowledge
You want to use the app even though you know the exercises and know what to do. So I turned it on every time even though I know how to do them.	Know the exercises and know what to do	Knowing what to do		
I think it has worked well. You see how you’re supposed to do the exercise and after a while you come to a conclusion how.	See how exercises are performed and come to a conclusion	Coming to the conclusion how	Developing reflection	
When I’m in the kitchen working you think about these things. That you can do it even though you don’t have a training session but you use the knowledge you gained.	Using the knowledge you gained	Using new knowledge		
		Identifying possibilities		

## Results

**Fig. 1:**
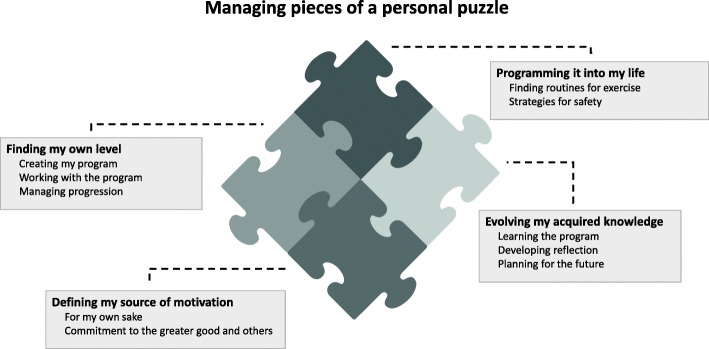
Overview of the theme and categories (bold in grey boxes) with corresponding subcategories

The analysis resulted in one theme, four categories and ten subcategories (Fig. 1). The theme ‘Managing pieces of a personal puzzle’ illustrates the participants’ experiences of independently being able to manage their exercise with the support of the program. This process of integrating the program in to their lives and in their way of thinking was flexible and shaped by their preferences and personal circumstances. The pieces in the puzzle are represented by the four categories, which encapsulates the participants’ experiences connected to self-management exercise programs. Managing the pieces represents several underlying processes of creating their own exercise program and finding new and safe routines for carrying it out. The processes were built on and carried forward by the participants’ motivation, their approach and their development of competence. The categories are further described below with their corresponding subcategories, together with quotations both interwoven and free-standing in italic (DP identifies those using the digital program and PB the paper booklet).

### Finding my own level

The category refers to how participants felt that the programs supported them in the process of finding their own level of exercise. Putting together an exercise program based on individual preferences and conditions, as well as finding and upholding the right intensity, is illustrated by the subcategories ‘Creating my program’, ‘Working with the program’ and ‘Managing progression’.

#### Creating my program

When the participants chose their own exercises and levels of difficulty at the start of the intervention, they selected exercises which they felt right for them, either by just looking through all the exercises or also by performing them. Finding the right level of the exercise was often described as a feeling that the exercise was just challenging enough and put as:*“…what I’ve experienced to be a bit difficult” (DP 8).* Choosing exercises was for the most part perceived as easy. However, for those with pain or medical problem this could sometimes set the limits for what exercises they felt were possible to choose, at all or on the particular day of training. Long experience of living with their medical condition gave them knowledge of what exercises suited them and when. They felt able to independently modify the exercises when they needed to, to make them more suitable for their bodily condition or simply because they wanted to: “*I can’t move my legs so far out when doing side-kicks. My hips are so stiff so I stop at a suitable height*” (PB 10). Participants using the digital program appreciated the flexibility of being able to choose between a great variety of exercises, which gave an opportunity to find exercises perceived as right for them*.*

*“I’ve taken out some exercises and replaced them with others. Some exercises you almost have to be a ballet dancer to be able to do so I’ve taken more, what should I call it, manlier exercises” (DP 16).*

#### Working with the program

Working with the programs was experienced generally as uncomplicated, with exercises which were easy to follow and were not overwhelming. For those using the booklet it was for the most part perceived as simply handled with clear instructions that were straightforward to understand.

*“You read, saw how many times you should perform it and you understood why you did it. It was easy and I believe that if it had been more complicated you wouldn’t have continued (PB 5).”*

However, sometimes the instructions on how to use the booklet to choose and organize the exercises was perceived as unclear, and more information was wanted.

Participants using the digital program perceived that the exercises were clearly explained as they could watch the videos and simultaneously hear the verbal instructions. Some, therefore, experienced the digital program as easier than paper-based programs that they had previous experience of.

*“I haven’t complied with the program [paper program] from the physiotherapist in the same way ….I think it’s because [in this digital program] it’s shown very clearly how to do it.”* (DP 3).

Participants in this group conveyed that it was pleasant to use the digital program and to be accompanied by the voice in the videos while exercising. It was perceived as encouraging and they felt they had “*a friend on the computer*” (DP 17).

#### Managing progression

Managing progression was described by the participants as developing their program by adding exercises at the beginning of the intervention and over time sorting out and replacing exercises that were too easy. It was considered to be important and motivating to feel strain while doing the exercises and a need for change occurred when they experienced the exercise as unchallenging.

*“M: After a few times we felt that it [the exercise] was so easy that it became no fun. W: We though, lets’ move on and change to a more difficult one!” (PB 3 + 4).*

The participants described different ways of managing progression. One way was to start at a low level, see how they got on with these, and then move on to new exercises when feeling a need to change to more challenging exercises.

*“I have progressed to more difficult exercises along the way, when I got used to them. I also did more repetitions than in the beginning and repeated the exercise.”* (PB 10).

Some identified a set of exercises after a while, which they thought were best suited for them or had become comfortable and familiar with, and therefore felt no further need for progression. Another way was to identify a few favourites, which could be described as core exercises, and changed the others from time to time. Some explained that they had changed exercises but could not describe how, when or why they had.

The participants experienced that the available exercises in the programs could set a limit for progression, when perceived as too easy. If so, some participants added a balance board or did more than the 10 exercises recommended, as strategies to achieve an enhanced experience of strain. Another strategy to increase the challenge, applied by booklet-users, was to do all the exercises in the booklet, often from the start of the intervention.

*“I wanted to try all exercises in the program so I would know how they felt. But, then I experienced improvement for each day, so I continued to do them all.”* (PB 11).

In comparison, users of the digital program described choosing the most difficult exercise in each group early on in the intervention, which led to diminished progression later on during the exercise period.

### Programming it into my life

Making room for the new exercise routines and programming them into their everyday lives, and finding a safe way of doing so, form important pieces of the participants’ personal puzzle. The participants’ experiences of self-management and independence when creating their own routines for exercise form this category which comprises the subcategories ‘Finding routines for exercise’ and ‘Strategies for safety’.

#### Finding routines for exercise

Finding routines for doing the exercise at home was described by the participants in both groups as an individual process of finding balance between flexibility and structure. The flexibility of being able to do the exercise in their own time and place was for the most part found to be positive.

*“It’s good to be able to exercise at home, you save time and you can do it whenever you feel like it without being directed.”* (DP 10).

The need for a clear structure was described by some and they followed a premade plan and did the exercises at a specific time each day, which could be put as: *“like I brush my teeth, I do this [the exercise routine]” (DP 16)*. Others did the exercise whenever it suited their other activities and appreciated the flexibility in this arrangement. Home exercises were for the most part experienced as positive, however the participants emphasized that group exercising could be perceived as more fun, social and committing and therefore also easier to get done.

When performing the exercise, different approaches were also described. One strategy was to do all exercises at once, believing it would create better effects by structurally following the program. Another approach was to spread the exercises over the day, either due to health issues or because they felt it made the exercise more enjoyable “…*I prefer it short and would rather repeat the exercise”* (DP 15). Some discussed integrating all, or some exercises, into their daily routines. Using integrated exercise was described to a lesser extent by the booklet-users, and if using this strategy it was often something they had learned already before this intervention, for example standing on one leg while brushing their teeth. Users of the digital program experienced the tips presented in the program for integration of exercises and for doing the exercises outdoors as positive. These tips were described as a help to develop and alter the activities they were already doing.

Participants in both groups described that discipline was required to follow their routines. When going away on holidays or having visitors it was easy to forget or skip exercising. To be reminded the booklet or the tablet could be left visible in strategic places, such as the kitchen table, and seeing it motivated them to do the exercise.

*“Of course you can have your phone ring when it’s time for exercise. But then you think: “not right now!” and you turn it off. I thought it was better having it [the booklet] laying in plain sight so you could see it.”* (PB 11).

#### Strategies for safety

The experienced need for safety varied among the participants in both groups and different strategies for performing the exercises safely were described. Overall, they expressed having well thought-out places in their home where the different exercises could be performed safely. Having hand held support or to be near support could often be perceived as essential, for some due to a fear of falling. Before training these participants identified safe surroundings or created those by rearranging furniture, and this strategy was never abandoned completely.

*“You have to think about safety. I lose my balance now and then and have to grab hold of a wall or a table when walking by. I have to be close to something to hold on to when I exercise.”* (DP 11).

The need of support was by others described as shifting depending on the exercise performed and they trusted their own capability of assessing their needs, which could be put as: “*You feel if you need support or not” (PB 9*). Others were very confident in their ability to perform the exercises safely in their own home because they felt that the exercises did not challenge them to the extent of being put out of balance. To be safe during exercise was all about knowing ones’ limitations.

### Evolving my acquired knowledge

This category highlight a process of building on competence by learning, and sometimes by reflecting, when self-managing exercise. The participants expressed evolving their acquired knowledge based on their own circumstances, their personal beliefs, and their need for support. The three subcategories ‘Learning the program, ‘Developing reflection’ and ‘Planning for the future’ form this category, which is a central part of the puzzle when establishing an individual exercise routine.

#### Learning the program

In both groups, the participants described how, at the beginning of the intervention, they used the program to look at the exercises every time they did their training. After a while they had seen and done the exercises so many times that they had learned them, expressed by a user of the digital program as: *“I know this by heart now” (DP 10*). They could therefore put the device or booklet aside while training, still it was perceived as good to go through the digital version/booklet from time to time, and to be reminded how to perform the exercises correctly. Learning the exercises by heart was more commonly described in the group using the digital program. In some interviews it was mentioned as better to learn the exercises in their program to avoid having to start up the computer or tablet.

*“In the beginning you had to look at the mobile phone every time you did the exercise. But then you learn, and you don’t have to look at every single exercise.”* (DP 7).

In contrast, other participants of both programs continued to look at the exercises every time they did their training as a way of maintaining a structured exercise session.

#### Developing reflection

The participants using the digital program described starting to reflect about the performance and difficulty of the exercises after doing them for a while. They could, for example, try different levels of support while doing the sit to stand exercise and reflect upon how strenuous it felt. Over time, by learning the exercises they learned what worked best for themselves and started to see new opportunities for training.

*“When I’m out walking I might see bicycle tracks in the snow and then follow them [tandem walk], because that’s balance too.”* (DP 6).

Reflections could also be facilitated by physical experiences. The participants expressed feeling similarities between the exercises and other activities such as climbing the stairs “*It’s activities you already do, but now I realise and feel that I exercise*” (DP 15). They then translated the exercises into their everyday lives and into new surroundings.

*“I realised I used a chair that was too high. I was going to get up after watching TV and noticed that it was really difficult. The sofa is quite comfortable and tilted back so you really have to make an effort. I then tried to exercise [doing sit-to-stand] from the sofa, as well as to put my feet together and that also went well.” (DP 2).*

Reflection and translation was expressed to a lesser extent by booklet-users, some described testing the exercises in new environments towards the end of the intervention.

#### Planning for the future

The participants in both groups presented different views of how they planned to continue with their training after the study. The need to continue to exercise regularly became particularly apparent for those who experienced benefits in relation to their medical conditions, or those who had felt major physical improvements during the study. The exercises presented in the programs could also be viewed as best suited during the winter as other activities, perceived as equally good exercise, became available during summer, for example gardening.

*“We need both strength and balance during the summer for the things we want to do, then you can’t dismiss all muscles during the winter. You have to do some maintenance work.”* (PB 10).

When planning for the future the participants spoke about doing the exercise in a more spontaneous way without obligation. This could mean choosing exercises that they had experienced as especially good and integrate them into their everyday activities or other physical activities such as daily walks or group training.

### Defining my source of motivation

The category presented in this section captures different motivational aspects for doing the exercise and participating in the study. Defining my own source of motivation, an important and personal piece of the puzzle, could be both internal ‘For my own sake’ and external as a ‘Commitment to the greater good and others’.

#### For my own sake

In order to maintain exercise routines it was important for participants in both groups to identify their own driving force for doing the exercise. Such motives could, for example, be a wish to remain strong and healthy, or the memory of an alarming situation in the past where they experienced a fall or had difficulties to manage independently in an everyday situation. Others described becoming more aware of their balance issues while doing the exercises in the program.

*“I was aware that I had a poor balance. But that it was that severe, as I discovered when starting to do these balance exercises, I had no idea.”* (DP 4).

The participants spoke about feeling pleased about their accomplishments and getting a good feeling in their body after doing the exercises, and thereby they developed a desire for doing the exercise. Motivation enhanced when they became aware of improvements in strength and balance or felt more secure during their daily walks. Such experiences gave a sense of importance to the exercise programs, and the exercises became more fun and inspiring to do. Attention to, and experiences of, improvements in strength and balance were more commonly expressed when using the digital program.

*“I’ve noticed that I’ve become better and that’s probably why I’m motivated and it feels important and fun.” (DP 17).*

#### Commitment to the greater good and others

The participants, in both groups, expressed that it was important to follow through on their commitment to the research project which a booklet-user described as: *“...if you have committed to this you cannot simply put it aside”* (PB 8). They described being aware of the societal problem of fall-related injuries and felt a responsibility to do their part in preventing falls and spreading awareness by contributing with information. The commitment to the project was more commonly expressed in the group using the booklet. Due to this commitment, participants sometimes continued to do the exercise despite health issues or feeling that the exercise was boring, and for some it became the key reason for getting the exercise done.

*“Honestly, I think exercise is dreadfully boring and I couldn’t write something in the exercise diary if I hadn’t exercised, so it was a must for me.”* (PB 1).

Some participants described that children or other relatives expressed worries about their stability and encouraged them to participate in the study. Those who joined the project together with their spouse described doing the exercise together and supporting each other. However, other participants expressed that they would have appreciated support from someone close.

“*M: We try to encourage each other. If you are a bit unmotivated to do the exercise you get helped by the other. W: I think it would have been much harder on your own.” (DP 7 + 8).*

Overall, the participants’ motivation was a central piece when managing the personal puzzle.

## Discussion

This study explores older people’s experiences of self-managed and self-tailored falls prevention exercise guided either by a digital program or a booklet. The main result, as presented in the theme ‘Managing pieces of a personal puzzle’, captures the participants’ experience of feeling competent to independently manage their exercise. Overall, the self-management approach enabled the older people in this study to tailor their exercise program to their own situation and preferences. This proved to be a process of creating their own personal puzzle through acquired knowledge, their motivation and the guidance of the program.

Putting together their own exercise program from a repository of evidence-based strength and balance exercises was, with the help of both programs, experienced as feasible. The participants appreciated the flexibility of being able to make decisions and choices that suited their own preferences and personal circumstances of bodily conditions and everyday activities. This suggests that the fall prevention exercise programs utilized in our study supported the process of self-tailoring. Self-tailoring means that the individuals who are taking part in a self-management intervention are able to utilize knowledge and skills of importance to a self-management intervention, and independently adopt it to their own situation [17]. These self-management skills have, within health care, previously been defined as problem solving, decision making, resource utilization, partnership with the health care provider, and taking action [17]. In the present study, these skills were commonly described by the participants, except partnership with a health care provider since the exercise was completely self-managed.

To date, there is little information about experiences regarding completely self-managed exercise interventions for older people. However, enabling older people to make own decisions regarding their exercise has previously been shown to improve health behaviours [31] and has also been suggested to be important for the uptake and adherence of fall prevention exercise [10]. Older people’s experience of participating in fall prevention programs also show that being involved in decision making as well as seeing the interventions as relevant and life-enhancing are valued components [32].

Our study shows that the participants tailored their exercise programs, the degree of manual support and their exercise environment, according to their experienced balance capacity. This is interpreted as a process in which they felt confident in assessing their limitations. However, one systematic review showed that 28% of the studies included reported that the older people did not want to exercise independently due to a fear of falling [14]. In this current study the participants were more confident in their ability to prevent a fall, possibly because they were provided with sufficient knowledge and support to feel secure and safe while exercising. Furthermore, our study shows that the participants valued the flexibility in being able to plan their exercise according to their everyday lives. Confirming this, previous research exploring views of older people in relation to a successful physical activity intervention showed that older people value flexible, affordable and accessible activities [33] and to be able to do the exercise in their own home [15].

Interestingly, as seen in the subcategory ‘Managing my progression’ there were many different ways of handling progression in the exercise programs. However, some described becoming comfortable with a set of exercises, or early on found favourites which led to diminished progression. Others described that the programs did not provide enough alternatives for progression. This implies that sufficient support for progression is important and future self-management programs should provide additional possibilities for progression, and more prompts or support regarding how and when to progress training.

The support that older people need to be able to exercise independently while feeling safe seems to have been provided by these self-management exercise programs together with the introduction meeting. Still, these completely self-managed exercise programs might not suit all older people. In some cases there might be a need for follow-up meetings with a physiotherapist to ensure sufficiently challenging exercises, or to provide external support to continue. This was exemplified by some participants using the booklet who described that their motivation to continue was only that the researchers would see the diary.

To increase development of new solutions to fall prevention exercise we need to challenge the concepts of traditional health professional-to-patient transition of information, where the health care provider is seen as the only expert. Alternative solutions could, through an empowering approach within the self-management treatment, support critical thinking and authorize the user to make informed decisions, and thereby increase patient autonomy [34]. Enhanced autonomy can, for example, be achieved by increased knowledge, development of skills and by the use of self-identified goals [35]. The two programs supported empowerment to different degrees. The different structures of the programs might have had an effect on how much the participants were able to adapt it to their personal circumstances and into new environments. The structure of the digital program made it easy to choose exercises from different levels of difficulty and suggestions about how to alter the exercises to make them more difficult or easy was provided in the videos. Furthermore, there were also suggestions of how to integrate exercises into daily activities and examples of outdoor activities.

Providing more guidance for self-management of exercise and behaviour change support in the digital program might also be one reason for the more commonly expressed learning and reflection when using the digital program. This could also be a result of the perceived ease to understand and choose exercises in this program. The video format made it possible to see the exercises being performed by older persons, in a home-environment, and simultaneously hear the verbal instructions. This also leaves less room for interpretation compared to images and text in standard paper programs. The videos might therefore have facilitated a transition of applying the knowledge gained, incorporating the exercises into their lives [17] and learning the exercises by heart. The digital program might more efficiently have empowered the participants to manage exercise on their own and thereby increased their self-efficacy. Reviews of empowerment-based digitally provided self-management interventions show promising results of enhanced empowerment, improved health status and increased physical activity [18, 36]. When empowering the patients to make their own decisions and behavioural changes, the actions become meaningful and persist over a longer period of time [37].

The gained competence, or mastering of an activity, has in previous research been shown to be a motivation for older people to continue exercising [14] and to experience personal benefits in independence and improved physiological and mental well-being [13, 14, 32]. This is also reflected in our study, in the subcategory ‘For my own sake’ where the participants expressed the importance of doing the exercise for themselves, feeling pleased about their accomplishments and seeing improvements. Turning points in self-management of diseases could be described as powerful emotional experiences or life changing insights [38] which has also been linked to increased internal motivation when self-managing type 2 diabetes [39]. Participants in this study described becoming aware of their balance issues, either by a fall or an everyday situation that they could not manage before entering the study, or improvements in physical functioning during participation. This could have created a turning point and increased their motivation for active participation in the study and the maintenance of exercise. Conversely, in the subcategory ‘For the greater good and others’ the commitment to the project and research was emphasised as a motivation for doing the exercise, which is also expressed by older people in previous research exploring views and preferences of exercise to prevent falls [13].

### Methodological discussion

This study explored the experiences of exercising with a self-management program guided by a digital program or a booklet. A strength in terms of *credibility* was the relatively large and varied sample, including participants from different recruitment settings, whereby we obtained different experiences and a richness of data [28]. Early during the analysis we could not see that those attending the peer-mentor meetings differed from the others in their experiences. Therefore a decision was made not to address in depth the experiences of attending the peer-mentor meetings.

To be able to conduct the many interviews, different interviewers with different levels of experience of conducting interviews were engaged. However, the varied experience could also be a strength as questions were asked from both novel and experienced perspectives, which we assume enriched the data and, in the next step, the interpretations. Several strategies were also used to enhance *transferability*.

A potential limitation is that one of the interviewers had met some of the participants during the introduction- and follow-up meeting. As this interviewer led some of the interviews herself, as well as supervised some of the students, it might have influenced the participants to leave more positive answers. The majority of the interviews were, however, conducted by researchers not involved in the main feasibility study.

A strength was that the codes, subcategories and theme on several occasions were deliberated upon to increase the *confirmability*. The researchers had a great variety regarding age, competences and perspectives (national/international, physiotherapy, exercise physiology, computing science, human-computer interaction), which in the analysis may have enhanced *credibility in* the process of triangulation between researchers [24]. All researchers were women and a variation of gender may have contributed with additional perspectives. The findings were, however, also validated though presentations at a research seminar and an international research meeting with researchers of varying competences, gender and ethnicity present, which broadened the perspectives on the analysis.

## Conclusion

Older people’s experiences of self-management falls prevention exercise guided by a digital program or a paper booklet were interpreted as ‘Managing pieces of a personal puzzle’. The flexibility and the independence provided by the programs when creating and finding routines for exercise was perceived overall as positive and constructive, which reinforces that self-management exercise is a feasible way of delivering fall prevention exercise. Different needs and preferences when managing their exercise were expressed as well as varying sources of motivation for doing the exercise. These findings present further insights that could aid in the design and implementation of fall prevention exercise programs that can be managed independently by the older person. A digital program seems to have supported learning and reflection to a higher degree than a booklet and might therefore create better opportunities for improved adoption and adherence in the long term. This study provides new knowledge regarding experiences, preferences and motivations of older people to engage in home-based self-managed fall prevention exercise.
